# Air Pollution and Parkinson Disease in a Population-Based Study

**DOI:** 10.1001/jamanetworkopen.2024.33602

**Published:** 2024-09-16

**Authors:** Brittany Krzyzanowski, Aidan F. Mullan, Pierpaolo Turcano, Emanuele Camerucci, James H. Bower, Rodolfo Savica

**Affiliations:** 1 Barrow Neurological Institute, Phoenix, Arizona; 2 Department of Neurology, Mayo Clinic, Rochester, Minnesota; 3 Department of Neurology, University of Kansas Medical Center, Kansas City; 4 Department of Quantitative Health Sciences, Mayo Clinic, Rochester, Minnesota

## Abstract

**Importance:**

The role of air pollution in risk and progression of Parkinson disease (PD) is unclear.

**Objective:**

To assess whether air pollution is associated with increased risk of PD and clinical characteristics of PD.

**Design, Setting, and Participants:**

This population-based case-control study included patients with PD and matched controls from the Rochester Epidemiology Project from 1998 to 2015. Data were analyzed from January to June 2024.

**Exposures:**

Mean annual exposure to particulate matter with a diameter of 2.5 µm or less (PM_2.5_) from 1998 to 2015 and mean annual exposure to nitrogen dioxide (NO_2_) from 2000 to 2014.

**Main Outcomes and Measures:**

Outcomes of interest were PD risk, all-cause mortality, presence of tremor-predominant vs akinetic rigid PD, and development of dyskinesia. Models were adjusted for age, sex, race and ethnicity, year of index, and urban vs rural residence.

**Results:**

A total of 346 patients with PD (median [IQR] age 72 [65-80] years; 216 [62.4%] male) were identified and matched on age and sex with 4813 controls (median [IQR] age, 72 [65-79] years, 2946 [61.2%] male). Greater PM_2.5_ exposure was associated with increased PD risk, and this risk was greatest after restricting to populations within metropolitan cores (odds ratio [OR], 1.23; 95% CI, 1.11-1.35) for the top quintile of PM_2.5_ exposure compared with the bottom quintile. Greater NO_2_ exposure was also associated with increased PD risk when comparing the top quintile with the bottom quintile (OR, 1.13; 95% CI, 1.07-1.19). Air pollution was associated with a 36% increased risk of akinetic rigid presentation (OR per each 1-μg/m^3^ increase in PM_2.5_, 1.36; 95% CI, 1.02-1.80). In analyses among patients with PD only, higher PM_2.5_ exposure was associated with greater risk for developing dyskinesia (HR per 1-μg/m^3^ increase in PM_2.5_, 1.42; 95% CI, 1.17-1.73), as was increased NO_2_ exposure (HR per 1 μg/m^3^ increase in NO_2_, 1.13; 95% CI, 1.06-1.19). There was no association between PM_2.5_ and all-cause mortality among patients with PD.

**Conclusions and Relevance:**

In this case-control study of air pollution and PD, higher levels of PM_2.5_ and NO_2_ exposure were associated with increased risk of PD; also, higher levels of PM_2.5_ exposure were associated with increased risk of developing akinetic rigid PD and dyskinesia compared with patients with PD exposed to lower levels. These findings suggest that reducing air pollution may reduce risk of PD, modify the PD phenotype, and reduce risk of dyskinesia.

## Key Points

**Question:**

Is air pollution in the form of particulate matter with a diameter of 2.5 µm or less (PM_2.5_) and nitrogen dioxide (NO_2_) associated with the risk and clinical characteristics of Parkinson disease (PD)?

**Findings:**

This case-control study including 346 patients with PD matched with 4813 controls found that PM_2.5_ and NO_2_ exposure was associated with statistically significant increases in PD risk and risk of developing dyskinesia. Higher exposure to PM_2.5_ was associated with a statistically significant increase in risk of the akinetic rigid PD subtype in subcohort analysis among patients with PD.

**Meaning:**

These findings suggest that a reduction in air pollution may help reduce PD risk, modifying the PD phenotype and the risk of dyskinesia in patients with PD.

## Teaser

This case-control study assesses the association of exposure to air pollution with risk of Parkinson disease and clinical characteristics among individuals with PD.

## Introduction

Parkinson disease (PD) is a degenerative disease that affects 2% of the population aged 70 years and older.^1^ The number of individuals with PD within the population is estimated to triple in the next 20 years.^2^ Several theories have been formulated to explain the progressive increase in the incidence of PD. Complex interactions among environmental factors, genetic predisposition, and known risk factors have been reported through the years as possible causes.^3,4^ Among environmental exposures, studies have suggested air pollution, in the form of aerosolized particulate matter with a diameter 2.5 µm or less (PM_2.5_), is associated with increased risk of PD.^5,6,7,8,9,10,11,12,13,14^ The ultrafine particles (≤0.1 µm) contained within PM_2.5_ may cross the blood brain barrier in humans,^15^ leading to inflammation, oxidative stress, and microglia activation, which are potential pathogenic mechanisms for the development of PD.^15,16,17,18,19^ At this time, there are no available national datasets on ultrafine particles contained in traffic pollution; however, ultrafine particles are traffic-related pollutants,^20^ along with nitrogen dioxide (NO_2_), for which nationwide data exist.^21^ Additionally, previous studies have implicated NO_2_ exposure as a PD risk factor.^22^ Thus, assessing the association of PM_2.5_ and NO_2_ with PD may help provide insight into the roles of different sources of air pollution in PD risk. In addition to potentially increasing the risk of developing PD, we hypothesize that air pollution exposure may also be associated with phenotypical manifestations and treatment outcomes. To our knowledge, no studies have explored the association between PM_2.5_ exposure and clinical phenotypes of PD. For this reason, we conducted a population-based study using data from the Rochester Epidemiology Project (REP) medical records linkage system to explore the association between PD and air pollution exposure. We also studied the association of air pollution exposure with patient mortality, different clinical characteristics, and presence of dyskinesia.

## Methods

### Study Consent

This case-control study was granted an exemption from review and informed consent by the Mayo Clinic institutional reviewer board. All patients and controls had Minnesota research authorization for use of medical records. This study is reported following the Strengthening the Reporting of Observational Studies in Epidemiology (STROBE) reporting guideline.

### Assessment of PD

We identified patients with PD in Olmstead County, Minnesota, from 1991 to 2015 using *International Classification of Diseases, Ninth Revision* (*ICD-9*) (332.0, 333.0, 331.82) and *International Statistical Classification of Diseases and Related Health Problems, Tenth Revision* (*ICD-10*) (G20, G21, G23.1, G23.2, G31.83) codes within the Rochester Epidemiology Project (REP) medical records linkage system.^23^ The records of all patients identified by *ICD-9* and *ICD-10* codes were reviewed by a movement disorder specialist (R.S.) to confirm the diagnosis of PD and determine the date of motor symptom onset. Cognitive symptoms were also reviewed for the diagnosis of PD. Details regarding the methods have been reported elsewhere.^23^ Although patients with PD were required to be living in Olmsted County at diagnosis date, they were not required to have lived in Olmsted County before that date. Therefore, our analysis includes patients with PD who lived outside of Olmsted County during the exposure window of interest (10 years prior to the date of PD symptom onset), and exposures were linked based on their prior addresses.

Controls were identified from the 27-county REP region in Minnesota, Iowa, and Wisconsin.^24^ Controls were screened for the same *ICD-9* and *ICD-10* codes for PD as were used to identify patients with PD in the case cohort. Controls were matched (using a randomly sorted greedy algorithm) to patients with PD 20:1 on sex and age within an index date that was 3 years prior to motor symptom onset for the matched patient with PD. All controls were required to not have any *ICD-9* or *ICD-10* codes for PD prior to the index date or up to 5 years after to ensure that no control developed PD motor symptoms. Patients with PD were divided into 2 subgroups (akinetic rigid and tremor-predominant PD subtypes) according to their most prominent feature on examination.^23^ Due to a low number of patients with tremor-predominant PD in the cohort, patients presenting with rest tremor and either bradykinesia or rigidity were considered tremor predominant in the analysis.

### PM_2.5_ and NO_2_ Exposure Estimation

Mean annual PM_2.5_ exposure data were collected from 1998 to 2019 from the Washington University in St Louis Atmospheric Composition Analysis Group.^25^ In addition, mean annual nitrogen dioxide (NO_2_) exposure data were collected from 2000 to 2014 from the Socioeconomic Data and Application Center.^26^ The PM_2.5_ and NO_2_ values for each patient and control were identified each year up to 10 years prior to the index date based on the 1-km^2^ area containing their home address of residency each year. Patients with PD with missing data for all 10 years before the index date were excluded, along with their corresponding matched controls.

As a sensitivity analysis, we restricted our patient population to metropolitan cores. In doing so, we ensure that our cases and controls were more comparable in terms of the spectrum of pollution they might have been exposed to. Metropolitan populations were defined as those living in a Rural Urban Commuting Area (RUCA) classification of metropolitan area core (RUCA = 1).

### Outcomes

Our primary outcome was risk of incident PD. Secondary outcomes were assessed only among patients with PD and included all-cause mortality following PD symptom onset, presence of tremor-predominant vs akinetic rigid PD, and development of dyskinesia.

### Statistical Analysis

We included 2 study designs: a case-control study design to assess the association of PM_2.5_ exposure with incidence of PD and a cohort study design focusing on PD subtypes and outcomes (dyskinesia and mortality) within our case group. All statistical analyses were performed during the January to June 2024. *P* values were 2-sided, and statistical significance was set at *P* ≤ .05. All analyses were conducted using R software version 4.2.2 (R Project for Statistical Computing).

#### Case-Control Study

In our case-control study, we modeled exposure in quintiles and using 2 linear splines, similar to prior studies of PM_2.5_.^5^ The placement of the knot was determined using bootstrap sampling to maximize the area under the receiver operating characteristics curve. Logistic regression was used with PD as the outcome and PM_2.5_ (or NO_2_) as the risk factor, adjusting for age, sex, race, ethnicity, year of index, and residency RUCA. All race and ethnicity information was taken directly from categories used in medical records. The other race category was reported directly in the medical record and not otherwise defined. We adjust for demographics that are well-established risk factors of PD. We adjust for year of index to diminish the potential impact of historical cohort effects. We adjust for RUCA to diminish the impact of differences that exist between urban and rural air pollution composition profiles. We further expect that our RUCA adjustment also diminishes the impact of differences that exist between urban and rural populations regarding other toxic exposures, including prior occupational exposures. RUCA designation was categorized as metropolitan area cores (RUCA = 1) and not metropolitan area cores (RUCA = 2-10). Results were reported as odds ratios (ORs) with 95% CIs.

#### Cohort Study

For our PD-specific cohort study of secondary outcomes, the risk of akinetic rigid subtype was assessed using logistic regression and the risk of all-cause mortality, and risk of dyskinesia was assessed using Cox proportional hazards regression. All models were adjusted for age, sex, race, ethnicity, and residency RUCA. Patient follow-up was censored at last available medical encounter or death, and PM_2.5_ was considered as a linear risk factor per 1 μg/m^3^. Model results were reported with ORs or hazard ratios (HRs) with 95% CIs. Differences in outcome based on PM_2.5_ exposure were highlighted using Kaplan-Meier cumulative incidence curves with PM_2.5_ divided into tertiles for patients with PD.

## Results

### Characteristics of Incident Cases

Of the 450 incident cases of PD identified from Olmsted County, 9 patients (2.0%) were excluded for missing address information and 95 patients (21.1%) were excluded for missing PM_2.5_ exposure data, resulting in 346 PD cases (76.9%; median [IQR] age 72 [65-80] years; 216 [62.4%] male) included for analysis, with 1 American Indian or Alaskan Native patient (0.3%), 6 Asian patients (1.7%), 5 Black or African American patients (1.4%), 1 Hawaiian or Pacific Islander patient (0.3%), 330 White patients (95.4%), and 3 patients identifying as other race (0.9%); 7 patients identified as Hispanic or Latino (2.0%) and 339 patients identified as not Hispanic or Latino (98.0%). Among 6920 controls matched to these included PD cases, 1875 (27.1%) were excluded for missing address information and 232 (3.4%) were excluded for missing PM_2.5_ exposure data, for a total of 4183 controls (69.6%; median [IQR] age, 72 [65-79] years, 2946 [61.2%] male), including 9 American Indian or Alaskan Native individuals (0.2%), 49 Asian individuals (1.0%), 33 Black or African American individuals (0.7%), 1 Hawaiian or Pacific Islander individual (<0.1%), 4164 White individuals (86.5%), 69 individuals identifying as other race (1.4%), and 488 individuals with unknown or undisclosed race (10.1%); 50 individuals identified as Hispanic or Latino (1.0%) and 4278 individuals identified as not Hispanic or Latino (88.9%). The median (IQR) time lived at these the current address was 15.9 (5.0-39.8) years. Most patients with PD lived inside metropolitan area cores (79.5%) compared with approximately one-third of controls (32.7%), which is why we include our metropolitan-restricted sensitivity analysis (Table 1; eTable in Supplement 1).

**Table 1.** Characteristics of Incident PD Cases and Controls

Characteristic	Participants, No. (%)	
	With PD (n = 346)	Controls (n = 4813)^a^
Age at index, median (IQR), y	72 (65-80)	72 (65-79)
Sex		
Female	130 (37.6)	1867 (38.8)
Male	216 (62.4)	2946 (61.2)
Race		
American Indian or Alaskan Native	1 (0.3)	9 (0.2)
Asian	6 (1.7)	49 (1.0)
Black or African American	5 (1.4)	33 (0.7)
Hawaiian or Pacific Islander	1 (0.3)	1 (<0.1)
White	330 (95.4)	4164 (86.5)
Other^b^	3 (0.9)	69 (1.4)
Unknown or did not disclose	0	488 (10.1)
Ethnicity		
Hispanic or Latino	7 (2.0)	50 (1.0)
Not Hispanic or Latino	339 (98.0)	4278 (88.9)
Unknown or did not disclose	0	485 (10.1)
RUCA classification		
Metropolitan core (RUCA 1)	275 (79.5)	1576 (32.7)
Not metropolitan (RUCA 2-10)	71 (20.5)	3237 (67.3)
Primary motor symptoms of PD		
Rest tremor	290 (83.8)	NA
Bradykinesia	316 (91.3)	NA
Impaired postural reflex	198 (57.2)	NA
Rigidity	295 (85.3)	NA
Medications taken for PD		
Levodopa	279 (80.6)	NA
Dopamine agonists	48 (13.9)	NA
Secondary outcomes		
Dyskinesia	54 (15.6)	NA
Death	259 (74.9)	NA

Abbreviations: NA, not applicable; PD, Parkinson disease; RUCA, Rural Urban Commuting Area.

^a^ PD cases and controls were matched on sex and age within 3 years.

^b^ Reported directly in the medical record and not otherwise defined.

### Risk of Parkinson Disease

Median (IQR) PM_2.5_ exposure prior to the index date was 10.07 (9.35-10.69) μg/m^3^ among patients with PD and 9.44 (8.69-10.22) μg/m^3^ among controls (Wilcoxon rank-sum *P* < .001). There was a positive association between PM_2.5_ and risk of PD: compared with the lowest quintile of PM_2.5_ exposure, the increased risk of PD associated with PM_2.5_ exposure ranged from 4% in the second quintile (OR, 1.04; 95% CI, 1.02-1.06) to 14% in the top quintile (OR, 1.14; 95% CI, 1.11-1.18) (Table 2). The median (IQR) NO_2_ exposure prior to the index date was 17.47 (15.46-19.99) μg/m^3^ for patients with PD and 17.17 (14.31-19.46) μg/m^3^ for controls (Wilcoxon rank-sum *P* = .27). There was a positive association between NO_2_ and risk of PD, but only for the top 2 quintiles of NO_2_ exposure. Compared with the lowest quintile of NO_2_ exposure, the odds of PD were increased by 5% in the fourth quintile (OR, 1.05; 95% CI, 1.01-1.10) and by 13% in the top quintile (OR, 1.13; 95% CI, 1.07-1.19) (Table 2).

**Table 2.** Association Between Mean Annual PM_2.5_ and NO_2_ Exposure and Risk of PD Using Logistic Regression

Air pollutant	Exposure, μg/m^3^, range	All PD cases and controls		Metropolitan populations only	
		OR (95% CI)^a^	*P* value	OR (95% CI)^b^	*P* value
PM_2.5_, quintile					
First (lowest)	5.05-8.55	1 [Reference]	NA	1 [Reference]	NA
Second	8.56-9.20	1.035 (1.01-1.06)	.002	1.10 (1.00-1.21)	.04
Third	9.21-9.77	1.07 (1.05-1.01)	<.001	1.15 (1.06-1.26)	.001
Fourth	9.78-10.48	1.10 (1.07-1.13)	<.001	1.17 (1.08-1.28)	<.001
Fifth (highest)	10.49-17.43	1.14 (1.11-1.18)	<.001	1.23 (1.11-1.35)	<.001
PM_2.5_, spline, per 1-μg/m^3^ increase					
1	5.05-10.60	1.05 (1.04-1.06)	<.001	1.05 (1.03-1.08)	<.001
2	10.61-17.43	1.01 (1.00-1.03)	.01	1.01 (0.98-1.05)	.47
NO_2_, quintile					
First (lowest)	4.64-13.57	1 [Reference]	NA	1 [Reference]	NA
Second	13.58-16.17	1.02 (0.97-1.06)	.46	1.02 (0.96-1.08)	.56
Third	16.18-18.25	1.02 (0.98-1.06)	.45	1.02 (0.96-1.08)	.54
Fourth	18.25-20.69	1.05 (1.01-1.10)	.03	1.02 (0.97-1.08)	.46
Fifth (highest)	20.70-48.51	1.13 (1.07-1.19)	<.001	1.11 (1.04-1.19)	.002

Abbreviations: NA, not applicable; NO_2_, nitrogen dioxide; OR, odds ratio; PD, Parkinson disease; PM_2.5_, particulate matter with a diameter ≤2.5 µm.

^a^ ORs were adjusted for age, sex, race, ethnicity, index year, and residency Rural Urban Commuting Area classification.

^b^ ORs were adjusted for age, sex, race, ethnicity, and index year.

**Figure 1.**
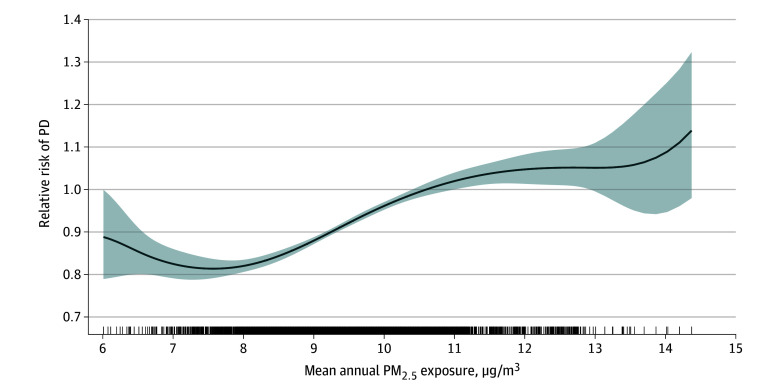
Risk of Parkinson Disease (PD) vs Controls by Mean Annual Exposure to Particulate Matter With a Diameter of 2.5 µm or Less (PM_2.5_) Relative risks are calculated compared with mean annual PM_2.5_ exposure of 10.6 μg/m^3^. Tick marks indicate distribution of exposure in the total sample; shading, 95% CI.

The trend in odds ratios across PM_2.5_ exposure was positive and linear with some tapering at the higher levels (Figure 1). This was observed in linear splines with a regression knot optimized at 10.6 μg/m^3^, with a 4.9% increase per 1-μg/m^3^ increase in PM_2.5_ exposure (OR per 1-μg/m^3^ increase, 1.05; 95% CI, 1.04-1.06) up to the knot at 10.6 μg/m^3^ and then a 1.7% increase per 1 μg/m^3^ above the knot (OR, 1.02; 95% CI, 1.00-1.03). A likelihood ratio test comparing the spline model to a linear model favored the nonlinear spline for modeling risk of PD (*P* < .001).

### PM_2.5_ Exposure on PD Subtype

Overall, there was a significant association between PM_2.5_ exposure and the development of akinetic rigid PD (Table 3). After accounting for patient age, sex, and residency RUCA classification, increased PM_2.5_ exposure was associated with a 36% increased risk of akinetic rigid presentation (OR per 1-μg/m^3^ increase, 1.36; 95% CI, 1.02-1.80, *P* = .03). The median (IQR) annual PM_2.5_ exposure for patients with tremor-predominant PD was 9.98 (9.31-10.65) μg/m^3^, compared with 10.51 (9.90-10.83) μg/m^3^ for patients with akinetic rigid PD.

**Table 3.** Association Between Average Annual PM_2.5_ Exposure and Risk of Parkinson Disease by PD Subtype Using Logistic Regression

Exposure	PM_2.5_ exposure, μg/m^3^, range	OR (95% CI)^a^	*P* value
**Tremor-predominant PD and matched controls (n = 4353)**			
Quintile			
First	5.05-8.55	1 [Reference]	NA
Second	8.56-9.20	1.06 (1.03-1.08)	<.001
Third	9.21-9.77	1.12 (1.09-1.15)	<.001
Fourth	9.78-10.48	1.17 (1.14-1.20)	<.001
Fifth	10.49-17.43	1.23 (1.19-1.27)	<.001
Spline, per 1-μg/m^3^ increase			
1	5.05-10.60	1.07 (1.06-1.08)	<.001
2	10.61-17.43	1.02 (1.00-1.04)	.02
**Akinetic-rigid PD and matched controls (n = 811)**			
Quintile			
First	5.05-8.55	1 [Reference]	NA
Second	8.56-9.20	1.06 (0.99-1.14)	.09
Third	9.21-9.77	1.08 (1.01-1.16)	.03
Fourth	9.78-10.48	1.12 (1.04-1.20)	.002
Fifth	10.49-17.43	1.24 (1.15-1.34)	<.001
Spline, per 1-μg/m^3^ increase			
1	5.05-10.60	1.07 (1.05-1.10)	<.001
2	10.61-17.43	1.04 (0.998-1.09)	.06

Abbreviations: NA, not applicable; OR, odds ratio; PD, Parkinson disease; PM_2.5_, particulate matter with a diameter ≤2.5 µm.

^a^ ORs were adjusted for age, sex, race, ethnicity, and index year.

### All-Cause Mortality and Dyskinesia

Among 346 patients with PD included in the study, 259 (74.9%) were deceased at the time of data abstraction, with a median (IQR) of 9.0 (6.0-11.8) years from PD symptom onset to death. After accounting for patient demographics (age, sex, race, and ethnicity) and RUCA, there was no significant association between level of PM_2.5_ exposure and mortality risk (HR per 1-μg/m^3^ increase, 0.93; 95% CI, 0.82-1.05; *P* = .23).

**Figure 2.**
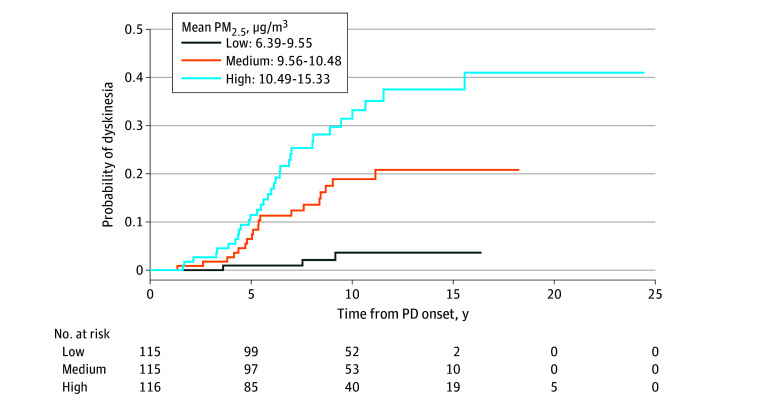
Kaplan-Meier Cumulative Incidence of Dyskinesia Among Patients With Parkinson Disease (PD) by Tertiles of Exposure to Particulate Matter With a Diameter of 2.5 µm or Less (PM_2.5_)

A total of 54 patients with PD (15.6%) developed dyskinesia at any time during the disease course. The median (IQR) time from PD symptom onset to dyskinesia was 5.6 (4.4-7.9) years. The Kaplan-Meier cumulative incidence for dyskinesia is shown in Figure 2, with PM_2.5_ classified by tertiles (high, medium, low). After accounting for patient demographics and RUCA, each 1-μg/m^3^ increase in PM_2.5_ was associated with 42% greater risk for developing dyskinesia (HR, 1.42; 95% CI, 1.17-1.73; *P* < .001).

### Sensitivity Analysis

Our analysis restricted to metropolitan core populations provided larger estimates compared with our primary analysis that included both metropolitan and nonmetropolitan populations (Table 1). In metropolitan populations, there was a positive association between PM_2.5_ exposure and PD risk. Compared with the lowest quintile of PM_2.5_ exposure, metropolitan populations had 10% to 23% increased odds of PD (second quintile: OR, 1.10; 95% CI, 1.00-1.21; fifth quintile: OR, 1.23; 95% CI, 1.11-1.35) (Table 2). However, our metropolitan-restricted analysis of dyskinesia (274 patients with PD; 37 dyskinesia events) found a lower risk compared with the analysis that included both metropolitan and nonmetropolitan populations. Specifically, we found that each 1-μg/m^3^ increase in PM_2.5_ was associated with 35% greater risk for dyskinesia (HR, 1.35; 95% CI, 1.06-1.72; *P* = .01) after accounting for patient demographics.

## Discussion

In this population-based case-control study, PM_2.5_ exposure was associated with an increased risk of developing PD, particularly the akinetic-rigid phenotype, and risk was higher with increasing PM_2.5_ levels. Exposure to NO_2_ was also associated with an increased risk of developing PD. Additionally, higher levels of PM_2.5_ and NO_2_ were associated with an increased risk of developing dyskinesia following the onset of PD. Contrary to our hypothesis and prior research,^27^ PD mortality was not associated with PM_2.5_ exposure. We speculate that patients with PD in our study area may have better access to medical care compared to individuals with PD in the general population.

Several studies have reported an association between PM_2.5_ exposure and adverse neurological outcomes.^5,28,29^ The ultrafine particles (≤0.1 µm) contained within PM_2.5_ cross the blood brain barrier,^15^ and PM_2.5_ in particular has been reported to be associated with inflammation, oxidative stress, and microglia activation, which are potential pathogenic mechanisms for the development of PD.^15,16,17,18,19^ Moreover, studies have demonstrated that higher levels of PM_2.5_ result in greater neurotoxic effects.^30^ Similar to other studies,^5,6,11,31,32^ we observed that the association between PM_2.5_ and PD risk tapered off at the highest levels of PM_2.5_. The reason for this plateauing remains unclear; however, some researchers have suggested that differences in PM_2.5_ composition in high-PM_2.5_ and low-PM_2.5_ regions may account for these findings_._^5^ Specifically, PM_2.5_ composition may be more heterogeneous in regions with the relatively high PM_2.5_, making PM_2.5_ alone a less reliable indicator of exposure to specific neurotoxic subcomponents in those regions. Nevertheless, we also acknowledge the possibility that the observed ceiling effect might be tied to a potential biological limit on the mechanisms of neuronal damage occurring in individuals chronically exposed to higher levels of PM_2.5_.

It is possible that PM_2.5_ may have varied effects on the development and progression of neurodegenerative disease based on its composition. A multicountry study in Europe confirmed the importance of considering the subcomponents of PM_2.5_.^7^ In our study, we were unable to explore broader ranges of PM_2.5_, since the range of PM_2.5_ in our study area (parts of Minnesota, Wisconsin, and Iowa) was relatively small compared with the range of PM_2.5_ observed nationwide. However, a 2022 study^33^ identified notable geographical variation of PM_2.5_ subcomponents in the Midwest, finding a north-south gradient in PM_2.5_, nitrite, and organic carbon composition, as well as an inverse gradient of sulfate composition. Additionally, the detected association with NO_2_ and the larger effect size observed in metropolitan core populations suggest the possibility that the PM_2.5_ association may be driven by traffic-related particulates. Unfortunately, without complete information of prior toxic exposures, we are limited in our ability to draw causal conclusions.

Importantly, in 2024, the US Environmental Protection Agency reduced the annual PM_2.5_ standard from 12 μg/m^3^ to 9 μg/m^3^ due to growing evidence of negative health effects at levels below the previously set standard.^34^ Our study not only supports the findings that led to this change, but suggests that the upper limit should be lowered to 8 μg/m^3^—a level previously advocated for by the American Lung Association and other health organizations. Notably, the World Health Organization recommends a more stringent limit than this, setting their standard to 5 μg/m^3^.^35^

Individuals with PD who were exposed to higher levels of PM_2.5_ were more likely to develop the akinetic rigid subtype of PD. Bradykinesia and rigidity are the predominant findings in these individuals, and this subtype has been linked to faster disease progression. Studies suggest that akinesia and resting tremor may result from different neurobiological processes, with the former resulting from both tonic (sustained) and phasic (intermittent) dopamine levels, and the latter from tonic release of dopamine and dopamine receptor responsiveness.^36^ We speculate that these differences may result from differences in PM_2.5_ subcomponents and subfractions. Interestingly, similar findings have been reported when using the neurotoxin 1-methyl-4-phenyl-1,2,3,6-tetrahydropyridine (MPTP) as a model for PD.^37^ Indeed, MPTP has been shown to produce both phasic and tonic dopamine dysregulation in the basal ganglia.^38,39,40^ In humans, MPTP can produce all major Parkinsonian symptoms, including akinesia and rest tremor; however, in many primate models, MPTP produces akinesia and rigidity without low-frequency tremor.^41^ Although MPTP is not found naturally in the environment, it is often referenced when exploring the role of environmental toxins that might cause neurodegeneration by a mechanism similar to MPTP.^42^ Thus our finding that PM_2.5_ exposure was associated with greater risk of the akinetic rigid PD subtype aligns with the possible evidence of a different clinical manifestations of the disease secondary to an external neurotoxin exposures (MPTP).^41^ This work provides insight into the role of PM_2.5_ exposure in the development of the different PD phenotypes. Furthermore, our study may offer a new explanation for the onset of dyskinesias that is not solely based on patient demographics, genetics, clinical characteristics, or drug response.^43,44,45,46^ In fact, it possible that environmental factors may lead to an increased risk of developing dyskinesia.

### Strengths and Limitations

Our study has several strengths. First, we used population-based incidence data, which allows us to better answer questions of PD etiology. Second, rather than relying on *ICD-9* and *ICD-10* codes alone, all identified cases were screened by a movement disorder specialist to confirm diagnosis of PD. Third, we used address-level data to assign exposure, which is a stronger proxy for patient-level PM_2.5_ exposure compared with less precise geographies (eg, zip codes or census tracts). Fourth, our REP data also allowed us to assign PM_2.5_ and NO_2_ exposure based on multiple years of address information for each patient, meaning that we were able to follow our patients forward through time.

This study also has some limitations. Our population-based dataset had a limited geographical extent. However, the REP captures data from patients for all health systems within our study area, making it a comprehensive population-based dataset.^24^ Our study was limited in that the REP population is predominantly White, given the demographics of the study region; however, our results reflect what other studies have found using diverse cohorts, including the nationwide Medicare population.^5^ We acknowledge that in our subtype analysis of PD cases, the distribution of PM_2.5_ among our PD cases was relatively small. Additionally, we were unable to adjust for all additional clinical characteristics associated with dyskinesia (eg, body weight, disease severity, and levodopa treatment). We did not have information on occupational history, work address, or activity space information; therefore, our results may be vulnerable to exposure misclassification errors (eg, for patients who spend more time at locations other than their home address). Long-term neurotoxic exposures are likely key in PD development. Due to the long prodromal period of PD,^47^ we used PM_2.5_ estimates for up to 10 years prior to symptom onset date. The relevant exposure window may extend back further, but PM_2.5_ estimates prior to 1998 are unavailable. Additionally, a limitation of many epidemiological studies is the use of clinical criteria that do not necessarily correlate with pathology findings and, usually, do not consider the presence of copathology. Importantly, it is possible that the toxicant role of PM_2.5_ may interfere with a change in the pathology cascade. On the other hand, we previously reported an clinicopathology concordance of 86.7% synucleoinopathies, supporting our case identification and classiffication.^48^

## Conclusions

This population-based case-control study provides evidence in support of an association of PM_2.5_ and NO_2_ exposure with the risk of developing PD. Higher levels of PM_2.5_ exposure were associated with increased risk of developing akinetic rigid disease and dyskinesias compared with lower levels of exposure. These findings suggest that a reduction in PM_2.5_ may help reduce the risk of PD and affect the clinical profile of PD and disease complications (modifying the PD phenotype and the risk of dyskinesia in patients with PD).

## Notes

Supplement 1. **eTable.** Frequency of Tremor-Predominant and Akinetic Rigid PD Subtypes by Average PM_2.5_ Exposure Prior to PD Symptom Onset

Supplement 2.
Data Sharing Statement
