# Supplementary material for: Air Pollution and Parkinson Disease in a Population-Based Study
Source: JAMA Netw Open. 2024 Sep 16;7(9):e2433602. doi: 10.1001/jamanetworkopen.2024.33602 (PMC11406396; doi:10.1001/jamanetworkopen.2024.33602)
Supplement: Supplement 1. — eTable. Frequency of Tremor-Predominant and Akinetic Rigid PD Subtypes by Average PM2.5 Exposure Prior to PD Symptom Onset [file jamanetwopen-e2433602-s001.pdf]

Supplemental Online Content

Krzyzanowski B, Mullan AF, Turcano P, Camerucci E, Bower JH, Savica R. Air pollution and Parkinson disease in a population-based study. *JAMA Netw Open*. 2024;7(9):e2433602. doi:10.1001/jamanetworkopen.2024.33602

eTable. Frequency of Tremor-Predominant and Akinetic Rigid PD Subtypes by Average $PM_{2.5}$ Exposure Prior to PD Symptom Onset

This supplemental material has been provided by the authors to give readers additional information about their work.

© 2024 Krzyzanowski B et al. *JAMA Network Open.*

eTable. Frequency of tremor-predominant and akinetic rigid PD subtypes by average $PM_{2.5}$ exposure prior to PD symptom onset

Average $PM_{2.5}$ Exposure	Total PD Patients	Tremor-Predominant	Akinetic Rigid
Under 9 $\mu g/m^3$	44	41 (93.2%)	3 (6.8%)
9-10 $\mu g/m^3$	119	107 (89.9%)	12 (10.1%)
10-11 $\mu g/m^3$	134	105 (78.4%)	29 (21.6%)
11 $\mu g/m^3$or higher	47	37 (78.7%)	10 (21.3%)
Overall	344	290 (84.3%)	54 (15.7%)

© 2024 Krzyzanowski B et al. *JAMA Network Open.*
