# Supplementary material for: Air Pollution and Parkinson Disease in a Population-Based Study
Source: JAMA Netw Open. 2024 Sep 16;7(9):e2433602. doi: 10.1001/jamanetworkopen.2024.33602 (PMC11406396; doi:10.1001/jamanetworkopen.2024.33602)
Supplement: Supplement 2. — Data Sharing Statement [file jamanetwopen-e2433602-s002.pdf]

Data Sharing Statement

Krzyzanowski. Air Pollution and Parkinson Disease in a Population-Based Study. *JAMA Netw Open*. Published September 16, 2024. doi:10.1001/jamanetworkopen.2024.33602

Data
Data available: No

Additional Information
Explanation for why data not available: Data is protected health information and cannot be shared.
